# Health care providers’ knowledge, attitude and perceived stigma regarding tuberculosis in a pastoralist community in Ethiopia: a cross-sectional study

**DOI:** 10.1186/s12913-018-3815-1

**Published:** 2019-01-08

**Authors:** Bezawit Temesgen Sima, Tefera Belachew, Fekadu Abebe

**Affiliations:** 1 Department of Health Education and Behavioral Science, Institute of Health, Jimma University, P.O. Box 378, Jimma, Ethiopia; 2 Department of Population and Family Health, Institute of Health Science, Jimma University, P.O. Box 378, Jimma, Ethiopia; 3 Department of Community Medicine and Global Health, Institute for Health and Society, Faculty of Medicine, University of Oslo, P.O. Box 1130, Blindern, 0318 Oslo, Norway

**Keywords:** Attitude, Control, Health care workers, *Kereyu*, Knowledge, Pastoralists, Stigma tuberculosis

## Abstract

**Background:**

Tuberculosis (TB) remains the prime killer disease among infectious diseases. TB control depends on early case detection and treatment in a directly observed treatment short course (DOTS) programme. The success of DOTS depends on the ability of the health care system to identify and properly manage TB cases. The present study aims to assess healthcare provider (HCP) knowledge, attitude and perceived stigma regarding TB and perception about traditional healers.

**Methods:**

A descriptive cross sectional study was conducted among 108 HCPs using a semi-structured, self-administered questionnaire from September 2014 to January 2015. The study district has a high TB burden area with one district hospital, 4 health centres, and 18 health posts. All health facilities and HCPs available during the study period in the district were included in the study. Statistical software for social science (SPSS) version 22 and STATA version 14 were used to enter and analyse data, respectively.

**Results:**

The majority (64%) of the HCPs had poor overall knowledge regarding TB, and 67.6 and 57.6% had poor knowledge regarding TB diagnosis and nature of the disease, respectively. Moreover, most 66.7 and 55.6% of the HCPs had an unfavourable attitude towards TB and TB control systems, respectively. Slightly under half (49.1%) of the HCPs had a favourable attitude towards TB patients, and the majority (88.9%) had low perceived stigma. The majority (87.0%) of the HCPs indicated the importance of community involvement in TB control activity. Moreover, most (60.2%) of the HCPs showed willingness to collaborate with traditional healers (THs) on TB control activity.

**Conclusions:**

Healthcare workers’ knowledge gap and unfavourable attitude towards TB control systems reported in this study may cause poor TB care delivery. HCPs’ perception of the importance of community involvement in TB control and willingness to collaborate with THs on TB management could be an opportunity to strengthen the World Health Organization’s (WHO’s) component of End TB strategy through community engagement. Training and workshops could be used to address the knowledge gap and the unfavourable attitude regarding TB among HCPs.

**Electronic supplementary material:**

The online version of this article (10.1186/s12913-018-3815-1) contains supplementary material, which is available to authorized users.

## Background

Tuberculosis (TB) is the leading cause of death and results in ill health for approximately 10 million people each year from a single infectious agent, ranking above human immunodeficiency virus/acquired immune deficiency syndrome (HIV/AIDS) [1]. Ethiopia ranks among the 30 highest TB burden countries, with estimated incidence of 200,000 TB per 100,000 populations in 2015. It has fully integrated the World Health Organization (WHO) End TB strategy into the national TB prevention and care plan [2]. The WHO reported that more than 3 million people with TB are not accurately diagnosed by the health care system every year [3], and healthcare workers (HCWs) have been reported to play a major role contributing to the success of TB treatment and management [4].

In Ethiopia, TB treatment is provided free of charge at primary, secondary and tertiary levels of public health facilities. Primary health care is the main mode of health care delivery, and the first point of medical contact for most rural residents in the country and is delivered through health centres and health posts. The success of directly observed treatment short course (DOTS) depends on the ability of the health care system to identify and properly manage TB cases. This requires active involvement of the HCPs in TB diagnosis and management [5, 6]. TB treatment should include counselling regarding disease progress and the importance of adherence to treatment. Failure to do so may result in the spread of TB and development of multi-drug resistant bacteria [7].

However, some studies reported misconceptions and lack of knowledge about TB among HCWs. For instance, a study in Iraq showed that only 12.6% of HCWs believe that TB is caused by bacteria, and another study in South Africa showed 21% of HCWs believe in prayer as treatment for TB [8, 9].

In pastoralist communities in Ethiopia, in addition to poor knowledge and poor healthcare-seeking behaviour [10–12], poor access to modern health care facility and quality of care has been a great challenge in the TB control activity [13–15]. Delays in the diagnosis and treatment of TB have also been reported [16–18]. Moreover, the Ethiopian national health care system has identified health extension workers (HEWs) as the first contact point in rural settings, which is difficult to implement in pastoralist communities due to their different lifestyle compared to non-pastoralists [19].

Studies have suggested that TB knowledge, attitude and stigma could be improved through training for HCWs and HCPs [20–22].

However, to our knowledge, there are no published reports on the knowledge, attitude and practice, and stigma towards TB among HCPs of pastoralist communities in Ethiopia, where access to health care is limited [23] and cultural differences between HCPs and the community are reported to be a challenge in the primary health care delivery system [24]. Therefore, this study aims to assess HCPs’ knowledge, attitude and perceived stigma regarding TB and perception regarding collaboration with traditional healers (THs) on TB control in the *Kereyu* pastoralist community in Ethiopia.

## Methods

### Study area and population

#### Study area

This study was conducted in *Fentalle* (*Kereyu* pastoralist) Wereda (equivalent to district), located in the east Shoa zone of Oromia, in the southern part of the northern rift valley of Ethiopia. The area falls within an altitude range of 800–1100 masl. The total land area is 1170 km^2^ with a total population of 76,367; it is located 200 km east of the capital city, Addis Ababa. *Metehara* is the capital and administrative centre of the district. A detailed description of the study area is given elsewhere [10].

There are four health centres and 18 health posts for the entire population of the *Kereyu* District. There is one referral hospital in *Metehara* Sugar Corporation called Merti Hospital. There was a total of 65 HEWs and 46 other health professionals (clinical nurses, midwifes, environmental health workers and pharmacy technicians and laboratory technicians) in the district, excluding the HCPs working at Merti Hospital, which is not the administrative unit of the district health office but does provide services to the pastoralist community. The number of HEWs reported at the district health office includes those training for upskilling, travelling for workshops, not available due to maternity or sick leave as well as those in the process of transfer to other districts. We included all available HEWs at the health posts during the study period.

### Study design and sampling

We conducted a facility-based cross sectional study from September 2014 to January 2015.

Before the actual data collection, we identified all the health facilities in the district with the help of HEWs and identified the TB focal person responsible for coordinating the district’s TB prevention and care activities. We included all HCPs and HEWs in the district in the study. Those who were available during the study period were given a self-administered questionnaire to complete and return. If the HCPs reported insufficient time to return the questionnaire the same day, they were allowed to return it the next day.

### Measurements

Data were collected using semi-structured and self-administered questionnaires prepared in English as in previous similar studies [20, 21, 25] and translated to Amharic (the federal working language). We gave training to three experienced local coordinators and involved them in the facilitation of the data collection process with the principal investigator.

The survey contained 71 questions, with sections on sociodemographic characteristics, TB knowledge, attitude, perceived stigma regarding TB and perception of collaboration with traditional healers. The tools were pre-tested before the actual data collection to assess the comprehensibility of the questionnaire.

### Knowledge

Knowledge is defined as the fact or condition of knowing something with familiarity gained through experience or association [22]. The knowledge section had 24 questions and was divided into three sections: TB diagnosis (10 questions about signs and symptoms of susceptive TB, active TB and relapse TB), nature of the disease (12 questions about transmission, cause, factors in the spread of TB) and treatment duration (2 questions, one for intensive phase and one for the whole duration of TB treatment required). The correct (yes) response to each question was scored as one for a positive response, and incorrect (no/I don’t know) response was scored as zero for a negative response. The scores were added together to generate a knowledge score from 0 to 24 (including each sign and symptom mentioned and factors for the exposure of TB), and the overall score was dichotomized using a median of 18 as a cut-off value. Those who scored 18 and above were coded “1” for good overall TB knowledge, and those below 18 coded “0” for poor overall TB knowledge. Likewise, scores were generated for the two sub-scales of knowledge regarding TB (TB diagnosis and nature of the disease), and the sub-scale of knowledge was categorized as poor and good levels of knowledge.

The term ‘nature of the disease’ is used to summarize the response of the HCPs about the organ affected most by TB, transmission, cause, factors for the spread of TB and the public importance of TB in the community.

### Attitude

Attitude is defined as how people feel about certain subjects or issues [21]. The attitude section contained 10 questions addressing two sub sections: attitude towards TB patients (3 questions) and attitude towards TB control (7 questions). A 5-point Likert scale was used to obtain responses to these questions and was treated as a continuous interval variable for analysis. The overall score for attitude regarding TB, attitude towards TB patients and TB control system was obtained by computing the included items using the SPSS syntax Compute by summing included items and multiplying the sum by 5 (number of Likert points). The attitude score was not normally distributed, therefore, overall attitude score was dichotomized using the median score of 37 as a cut-off value; those who had a mean score of 37 and above were coded “1” for a favourable attitude regarding TB and below 37 coded “0”, indicating an unfavourable attitude regarding TB.

### Perceived stigma

Perceived stigma refers to the fear of discrimination or, in general, to the awareness of negative attitude and/or practices related to a particular condition [26]. The perceived stigma section had three sections: feeling about a person with TB, perceived community feelings towards TB patients and feelings about being near a person with TB. The first section had five items and was summed to create a perceived stigma score towards TB patients for analysis. Each item was coded as a “yes” or “no” response where ‘yes’ indicated the absence of perceived stigma and ‘no’ indicated the presence of perceived stigma. Negatively stated questions were reverse coded to obtain the correct scoring. The responses consistent with “lack of stigma” were scored one and the rest scored zero. The sum of the responses to (1) I feel compassion and desire to help; (2) I feel compassion but tend to stay away from TB patients; (3) It is their problem, and I cannot get TB; (4) I feel fear because they might infect me; and (5) I have no particular feelings were used to generate stigma score from 0 to 5. The overall score was dichotomized using the median as a cut-off point. Since the stigma scores were not normally distributed, the median score (median 1, IQR = 1) was used to classify the HCPs as having high or low perceived stigma towards TB patients. Those who have a score above 1 were coded as one, showing high perceived stigma towards TB patients, and those who scored 1 or lower were coded as zero, showing low perceived stigma towards TB patients (Additional file 1).

### Perception about THs

Perception is man’s primary form of cognitive contact with the world around him [27]. In this study, we assessed the HCPs’ perception for possible future collaboration of the conventional health system with THs on TB control. Perception of the HCPs regarding collaboration with THs and their willingness to collaborate on TB prevention and care was assessed using 17 items. The questions had multiple choice and ‘yes’ or ‘no’ responses. The proportion of responses to some of the items used and relationship to the conclusion of this paper is reported.

### Data analysis

#### Quantitative data

The data were entered and analysed using statistical software for social science (SPSS) version 22 and STATA version 14. We applied descriptive statistics to summarize the socio-demographic status of the HCPs, describe their knowledge and attitude and perceived stigma regarding TB and their perception about collaboration with THs on TB diagnosis and treatment. A Chi-square test was used in bivariate analysis to determine the association between the outcome variables and selected covariates. Univariate logistic regression was used to assess the strength of the association. The statistical significance of the differences was evaluated using *p* value < 0.05 and a 95% confidence interval.

## Results

In total, 108 HCPs participated in the study. Fifty-eight percent were male with most (63%) less than 30 years old (mean age 29). A large proportion of the study participants were nurses followed by health officers, accounting for 49.1 and 25.0%, respectively, while 22% were HEWs. Sixty-three percent of the HCPs do not have TB-related training, while 65% of the respondents had worked in the TB unit for more than 6 months (Table 1).

**Table 1:** Socio-demographic characteristics of HCPs in the *Kereyu* pastoralist district, Ethiopia

Variables	Frequency (*n* = 108)	Percent %
Age		
< 30	68	63.0
30–40	26	24.1
> 40	14	13.0
Sex		
Male	63	58.3
Female	45	41.7
Profession		
Medical doctor	3	2.8
BSc Nurses	53	49.1
Health officers	27	25.0
Pharmacists	1	0.9
HEWs	24	22.2
Work duration		
< 2 years	35	32.4
3–5 years	27	25.0
6 to 10 years	28	25.9
> 10 years	18	16.7
Work at the DOTS unit		
< 6 months	15	13.9
> 6 months	70	64.8
Never	23	21.3
Attended DOTS/TB training		
Yes	40	37.0
No	68	63.0
Provide health education on TB		
Yes	55	50.9

### Knowledge regarding TB

Table 2 shows the overall knowledge of the HCPs regarding TB and factors associated with this knowledge. Approximately 64% of HCPs had a poor level of knowledge about TB. Most of the HCWs also had poor knowledge of the nature of the disease (57.6%) and aspects of TB diagnosis (67.6%). HCWs who had worked for < 2 years, 6–10 years and > 10 years had better overall knowledge regarding TB by 4.3 and 4.6 points, respectively compared with HCWs with working duration of 3–5 years (*p* = 0.02, *p* = 0.03 and *p* = 0.03, respectively).

**Table 2:** Knowledge level and factors associated with knowledge regarding TB among HCPs in *Kereyu* pastoralist district, Ethiopia

Variable	Median (Q1,Q3)	Knowledge Level		
Knowledge category		poor NO. (%)	good NO. (%)	
Towards nature of the disease	6 (6,7)	62 (57.64)	46 (42.6)	
Towards TB diagnosis	10 (6,11)	73 (67.6)	35 (32.4)	
Overall TB knowledge	18 (14,19)	69 (63.9)	39 (31.6)	
		Knowledge about TB diagnosis		
Attending TB training	COR	Poor NO. (%)	Good NO. (%)	P
Yes	2.45	22 (30.0)	18 (51.4)	0.03
No	Ref.	51 (69.9)	17 (48.6)	–
		Knowledge about the nature of TB		
Duration of work at DOTS unit	COR	Poor NO. (%)	Good NO. (%)	
< 6 months	5.39	6 (9.7)	9 (19.6)	0.02
> 6 months	3.05	38 (61.3)	32 (69.6)	0.047
Never	Ref.	18 (29.0)	5 (10.9)	–
		Overall Knowledge regarding TB		
Duration of work at the health facility	COR	Poor NO. (%)	Good NO. (%)	
< 2 years	4.3	20 (29.0)	15 (38.5)	0.02
3–5 years	Ref.	23 (33.3)	4 (10.3)	–
6–10 years	4.3	16 (23.2)	12 (30.8)	0.03
> 10 years	4.6	10 (14.5)	8 (20.5)	0.03

*COR – crude odds ratio

*Q1 and Q3 – quartile 1 and 3

Attending TB training was associated with a 2.45-point increase in knowledge score about TB diagnosis compared with those who had never attended TB training (*p* = 0.03). Working at the DOTS units increased knowledge about the nature of TB (for less than 6 months yielded a 5.39-point increase and greater than 6 months with a 3.05-point increase, respectively) compared with those who had never worked at the DOTS unit (*p* = 0.02 and *p* = 0.047, respectively) (Table 2).

Regarding HCPs’ knowledge of the body organs most affected by TB (Table 3), the majority (89.7%) answered that TB affects the lungs, while 7.4, 1.9 and 0.9% reported that it affects the bones, kidney and abdominal organs, respectively. Moreover, 90.7% of the HCPs mentioned that the main risk factor for the spread of TB infection is close household contact with an active TB patient, while 5.6, 9.3 and 5.6% said that overcrowding, humidity and poor nutrition, respectively, was the risk factor for the spread of TB. In addition, 77.8% of the HCPs knew the correct definition of relapse TB (patient cured/completed treatment and return with smear positive sputum). Concerning the duration of TB treatment for new diagnosed active anti-pulmonary TB, 94.4% answered correctly (6 months) (Table 3).

**Table 3:** HCPs’ Knowledge regarding TB in *Kereyu pastoralist* district, Ethiopia

Variables	Frequency (*n* = 108)	Percent (%)		
Cause of TB				
Mycobacterium tuberculosis	106	98.1		
Mycobacterium pneumonia	1	0.9		
Mycobacterium contagiosum	1	0.9		
Organs most affected				
Lung	97	89.8		
Bones	8	7.4		
Kidney	2	1.9		
Abdominal organs	1	0.9		
Routes of TB transmission				
Droplets from coughing and sneezing of a person with active TB	94	87		
Sharing cups	13	12		
Handshaking	1	0.9		
Factors for the spread of TB				
Household contact	98	90.7		
Overcrowding	6	5.6		
Humidity	10	9.3		
Poor nutrition	6	5.6		
People at high risk of developing TB	Yes	Percent%	No	Percent %
HIV positive	94	87	14	13
People in contact with TB patient	46	42.6	62	57.4
People with chronic disease	28	25.9	80	74.1
Pregnant women	6	5.6	102	94.4
Infectious type of TB				
Active pulmonary TB	100	92.6		7.4
TB in other organs/body parts	8	7.4		1.9
Symptoms suspicious for TB				
Cough for more than three weeks	74	68.5	34	31.5
Fever	87	80.6	21	19.4
Haemoptysis	94	87	14	13
Night sweating	83	76.9	25	23.1
Loss of appetite	78	72.2	30	27.8
Loss of weight	77	71.3	31	28.7
General weakness	71	65.7	337	34.3
Chest pain	82	75.9	26	24.1
Diagnosis of Active PTB				
Two or three positive smear tests	95	88.0		
One positive smear and positive X-ray	13	12.0		
Relapse TB				
Completed treatment. Cured and returned with positive smear	84	77.8		
Under treatment & sputum remains positive after 5 months	10	9.3		
Interrupted treatment for 3 months and returned with positive smear	14	13		
Duration of active PTB treatment				
6 months	102	94.4		
9 months	3	2.8		
2–5 months	1	0.9		
Do not know	1	0.9		
Duration of intensive TB treatment phase				
2 months	94	87.0		
6 months	11	10.2		
9 months	1	0.9		
12 months	2	1.9		
MDR-TB				
When the bacilli is resistant to all currently available drugs of TB	32	29.6		
When the bacilli is resistant to at least isoniazid and pyrazinamide	49	45.4		
When the bacilli is very aggressive and you need at least 8 to 12 months of treatment	23	21.3		
Others	4	3.7		

*MDR-TB – Multi-drug resistant TB

### Attitude towards TB

Table 4 shows that 53.7% of the HCPs had an unfavourable attitude towards TB. A large proportion (55.6%) had an unfavourable attitude towards TB control systems. A large proportion (66.7%) of the HCPs had an unfavourable attitude towards TB patients. Those who believed that people with HIV are most affected by TB had approximately a 4 times more favourable attitude towards TB compared with those who did not mention people with HIV as a high risk group for TB (*p* = 0.027).

**Table 4:** Attitude Score towards TB and factors associated with overall attitude towards TB

Variable	Median score (Q1, Q3)	Attitude		
		Favourable NO. (%)	Unfavourable NO. (%)	
Towards TB control	26 (23,30)	48 (44.4)	60 (55.6)	
Towards TB patient	12 (10,13)	36 (33.3)	72 (66.7)	
Overall Attitude	37 (34,42)	53 (49.1)	55 (50.9)	
Overall Attitude towards TB				
	COR	People with HIV are most affected by TB Yes No No. % No. %		P
Favourable Unfavourable	4.1 Ref.	50 (53.2) 44 (46.8)	3 (21.4) * 11 (78.6)	0.027

COR – crude odds ratio

Q1 and Q3 - quartile 1 and 3

*Reference

Eighty-seven percent of the respondents strongly agreed with the statement that community engagement is essential for TB control, and a substantial proportion (44.4%) of the HCPs also strongly agreed that the service they provide is accepted in the community (Table 5).

**Table 5:** HCPs’ attitude towards TB in *Kereyu* pastoralist district in Ethiopia

Variables Frequency (*n* = 108)					
	Strongly agree	Agree	Neutral	Disagree	Strongly disagree
New cases of TB are major challenges for TB control	38 (35.2%)	33 (30.6%)	13 (12.0%)	15 (13.9%)	9 (8.3%)
Community involvement is important in TB prevention and control	94 (87.0%)	13 (12.0%)	1 (0.9)	0	0
TB patients do not understand why they should take the medication after starting to feel better	36 (33.3%)	51 (47.2)	9 (8.3%)	10 (9.3%)	2 (1.9%)
MDR-TB is a major public health problem in your community	58 (53.7%)	29 (26.9%)	5 (4.6%)	14 (13.0%)	2 (1.9%)
Using DOTS makes a difference in treatment compliance	25 (23.1%)	34 (31.5%)	22 (20.4%)	22 (20.4%)	5 (4.6%)
A person with TB faces stigma and shame in your community	27 (25.0%)	26 (24.1%)	18 (16.7%)	33 (30.6%)	4 (3.7%)
DOTS implementation should take individual circumstances in to consideration	32 (29.6%)	23 (21.3%)	9 (8.3%)	32 (29.6%)	12 (11.1%)
Poor knowledge about TB makes it difficult to follow DOTS	36 (33.3%)	52 (48.1%)	0	15 (13.9%)	5 (4.6%)
TB treatment we provide is accepted by the clients	48 (44.4%)	40 (37.0%)	12 (11.1%)	3 (2.8%)	5 (4.6%)
Most HCWs at this facility have adequate training for TB control activities	17 (15.7%)	23 (21.3%)	9 (8.3%)	44 (40.7%)	15 (13.9%)

### Perceived stigma towards TB patients

Regarding the perceived stigma of HCPs towards TB patients, the majority (88.9%) had low perceived stigma and few (11.1%) had high perceived stigma towards TB patients. Table 6 shows that overall knowledge of the HCPs towards TB is significantly associated with the perceived stigma of the HCPs towards TB patients (*p* < 0.001).

**Table 6:** Factors associated with perceived stigma towards TB patients in *Kereyu* pastoralist district, Ethiopia

Variables	Perceived stigma level		
Overall knowledge towards TB	Low perceived stigma No. (%)	High perceived stigma No. (%)	*P* value
Good knowledge	39 (40.6)	0 (00.0)	0.001
Poor knowledge	57 (59.4)	12 (100.0)	

Perceived stigma level categorized as high and low using median and interquartile range

The majority of the HCPs (75.9%) mentioned feeling compassion and a desire to help; 56.5% indicated that the community mostly supports TB patients (Table 7).

**Table 7:** HCPs’ Perceived stigma towards TB in *Kereyu* pastoralist district, Ethiopia

Variables	Frequency (*n* = 108)	Percent%
Feeling about a person with TB		
I feel compassion and a desire to help	82	75.9
I feel compassion but I tend to stay away from these people	8	7.4
I am afraid of them because they might infect me	4	3.7
I have no particular feeling	14	13.0
TB is a shameful disease		
Yes	33	30.6
No	75	69.4
How TB patients are regarded/treated in the community		
Most people reject him or her	23	21.3
Most people are friendly but they generally avoid him/her	32	21.3
The community mostly supports her/him	61	56.5
Others	1	0.9
Feeling around a person having TB		
I feel like I would get infected so I will make my conversation short	25	23.1
I feel like I have to keep my distance	9	8.3
I feel like I have to be supportive	73	67.6
Others (no particular feeling)	1	0.9

### Perception of HCPs towards collaboration with THs

Table 8 shows that the majority (60.2%) of HCPs are willing to work in collaboration with THs. Referral of potential TB patients by THs (29.8%) and cross visiting (29.9%) were among the possible methods of collaboration on TB prevention and care mentioned by HCPs.

**Table 8:** HCPs’ perception of collaboration with THs for TB control in *Keryu* pastoralists District, Ethiopia

Variables	Frequency (*n* = 108)	Percent%
Traditional medicine exist		
Yes	12	11.1
People in this community go to TH for TB treatment	48	44.4
Most preferred treatment option		
Traditional medicine	4	3.7
Modern medicine	24	22.2
Reason for traditional medicine preference	75	69.4
Easy accessibility	26	24.1
Traditionally acceptable	61	56.5
Less time taking	14	13
Others	7	6.5
Most Preferred to consult for health problems		
THs	37	34.3
Healthcare provider	71	65.7
Know a patient who visited healthcare facility soon after visiting THs for TB	30	27.8
Treated a person with TB referred by THs	19	17.6
Why TH do not refer TB patients		
TH can treat TB	17	15.7
THs fear losing patient trust	66	61.1
No collaboration mechanisms	52	48.1
THs fear losing money	40	37.0
No referral system	26	24.1
No trust in modern medicine	16	14.8
THs fear critics	2	1.9
Do not know	2	1.9
Others	7	6.5
Accept THs practice	19	17.6
Collaboration options		
Cross visiting	29	26.9
TH learning about TB	16	14.8
Working together	29	26.9
THs refer patients to healthcare facility	29	26.9
Training THs	2	1.9
Joint research programme	3	2.8

## Discussion

The current study assessed the knowledge, attitude and perceived stigma of HCPs regarding TB and perception of THs. The results show that the majority of the HCPs had poor overall knowledge regarding TB, with particularly poor knowledge about the nature of the disease and aspects of diagnosis. In addition, most of the HCPs had an unfavourable attitude towards TB and TB patients as well as the TB control system. Interestingly, most HCPs in this study had low perceived stigma towards TB patients.

The low level of knowledge regarding TB among HCPs in this study is in line with other studies examining communities with similar high TB burden and rural settings in African countries [28, 29] and other settings [30]. These results also indicated that attending TB training increased HCPs’ knowledge regarding TB diagnosis more than two-fold compared with those who did not have TB training. In addition, a longer duration of work and working for less than 2 years at the healthcare facility and experience working at the DOTS unit are significantly associated with increased knowledge regarding TB. This might indicate a lack of training and updating the HCPs with new and current national TB control guidelines in the district. HCPs’ knowledge regarding diagnosis and management of TB is crucial for TB control through proper case management, and it has a direct impact on the effectiveness and quality of the health care provided [7, 31, 32].

The majority of the HCPs in this study knew that bacteria cause TB. This is in contrast to studies in Iraq and South Africa, where misconceptions about TB among health care workers were more frequent. For instance, a study in Iraq [20] showed that only 12.6% of HCWs believed TB is caused by bacteria, and another study in south Africa showed 21% of HCWs believe in prayer as a treatment for TB [8].

In the present study, most HCPs had an unfavourable attitude towards TB, and most also demonstrated an unfavourable attitude towards TB patents and TB control system. Studies reported that the negative attitude of providers towards TB patients and the existing treatment system has led to a high treatment dropout rate, while home visits by providers and supportive and caring staff resulted in high treatment compliance and positive experience with DOTS [4, 30].

In the present study, the majority of the HCPs acknowledged the importance of community involvement in TB control activities and expressed their willingness to collaborate with THs on TB control activities. In this regard, many studies have reported positive contributions by THs to TB management through collaboration with modern medicine in resource-poor settings [25, 33].

Knowledge, attitude and perceived stigma of the HCPs regarding TB were not significantly associated with age, sex or profession of the HCPs. The low scores might also be because the questionnaires were administered to all HCPs in spite of their engagement at the DOTS clinic during the study period.

## Limitations

HCPs’ knowledge, attitude and perceived stigma regarding TB and their perception about THs in *kereyu* pastoralist district were assessed. To the best of our knowledge, no similar study has been conducted in *kereyu* pastoralist communities in Ethiopia. This could help to strengthen TB prevention and care activities in the district. However, this study has limitations because the report does not include TB case management/diagnosis and treatment practice of the HCPs at the TB unit. In addition, the self-reporting nature of the study and the fact that HCPs were allowed to return the completed questionnaire the next day might allow the respondents to receive external assistance in responding to the questions, affecting the results. Moreover, we were not able to include all HCPs reported to be working at the target healthcare facilities because of absence for training, workshops, leave of absence, transfer process, etc. Furthermore, the stigma questionnaire was not validated.

## Conclusions

This study provides relevant information about the HCPs’ knowledge gap regarding TB, unfavourable attitude towards TB and TB prevention and care system. Our results showed that the HCPs had an unfavourable attitude towards the TB control system and had low perceived stigma towards TB patients. Training and workshops could help to fill in the knowledge gap of the HCPs’ and change their attitude towards TB control system and TB patients. The HCPs interest in involving the community and THs in TB prevention and care activity is an opportunity to implement or strengthen the WHO’s End TB strategy through community engagement.

## Additional file

Additional file 1: Questionnaires (DOCX 40 kb)
