# Supplementary material for: Health care providers’ knowledge, attitude and perceived stigma regarding tuberculosis in a pastoralist community in Ethiopia: a cross-sectional study
Source: BMC Health Serv Res. 2019 Jan 8;19:19. doi: 10.1186/s12913-018-3815-1 (PMC6325851; doi:10.1186/s12913-018-3815-1)
Supplement: Supplementary file 1 — Questionnaires (DOCX 40 kb) [file 12913_2018_3815_MOESM1_ESM.docx]

Supporting File 1: Questionnaire

Self-administered questionnaires on Knowledge, attitude and perceived stigma of healthcare workers regarding TB

**I. socio demographic data**

1. Age in years:________________

2. Sex a. Male b. Female

- 1. Religion

1. Christian b. Muslim c. Other Please specify: _______________
   1. Place of birth____________
   2. How long have you worked in health facility (in months/years): __________
   3. In which health facility do you currently work? ____________________
   4. What is your profession currently?
2. Medical doctor b. BSC Nurse c. Health officer

d. 10 +3 (Health extension worker) e. Others please specify: _________

- 1. How long have you been working in this health care facility? (in months/years): ___________­­­­­_________________________
  2. How long have you worked at the DOTS clinic in this Health care facility?
     1. < 6 months
     2. > 6months
     3. Never
     4. Others please specify: ________________
  3. Have you ever received training on DOTS provision?
     1. Yes
     2. No
  4. Is providing Health education part of your duty at this health facility?
     1. Yes
     2. No
  5. Do you currently provide Health education on TB?
     1. Yes
     2. No
  6. If so how often do you provide education on TB?

1. Daily
2. Twice a week
3. Once a week
4. Others please specify: __________

13a. what materials do you use to guide your education?

- 1. IEC materials from ministry of health
  2. Books
  3. Video’s
  4. Others please specify: _____________
  5. What do you think would make it easier for communities to have more open dialogues on health topics?

1. To use religious leaders
2. Use community leaders
3. use local language
4. others please specify: ________________
   1. What do you think is the best communication channel that is likely to be effective in reaching the majority of TB behavior change targets in your community?
   2. Radio
   3. TV
   4. HEWs
   5. Others please specify: _______________

- 1. What do you think are the advantages of DOTS?

1. people can be closely monitored to take their anti TB drugs
2. can avoid default rate
3. insure high cure rate
4. help to know the outcome of the treatment
5. others please specify___________
6. What do you think could be the disadvantage of DOTS?
7. inconvenience
8. stigma from health provider
9. lack of social support
10. others please specify__________________

**Knowledge about Tuberculosis**

- - - 1. Which one of human organs could be affected by TB? (Check all possible ans.)

1. Lung
2. Bones
3. Kidney
4. Uterus
5. Abdomen
   - - 1. What is the germ that causes TB? (check only one)
   1. Mycobacterium tuberculosis
   2. Mycobacterium pneumonia
   3. Mycobacterium ayium
   4. Mycobacterium contagiousum
   5. Virus
   6. Others please specify: ____________
      - 1. What are the routes of TB transmission? ( you can check more than one)
6. Droplets during coughing and sneezing
7. Sharing cups
8. Handshaking
9. Others please specify: ______________

- - - 1. What do you think are the factors relevant to TB infection spread? ( you can check more than one)
  1. Household contacts
  2. Overcrowding
  3. Humidity
  4. Under nutrition
  5. Others please specify: ___________
     - 1. Which group of people is at high risk of developing TB? (Check all options)

1. People with HIV
2. People who have close contact with a person having TB
3. People with chronic disease
4. Pregnant
5. Others please specify_________________
   - - 1. Which type of TB do you think is high infection source for TB? (check only one)
   1. Active pulmonary TB
   2. TB in other organs
   3. Contaminated organs
   4. Other please specify: _____________

## Tuberculosis Diagnoses

- - - 1. What are the signs suspicious for TB? ( you can check more than one)

1. Cough ≥ 3 weeks
2. Fever
3. Haemoptysis
4. Night sweating
5. Loss of appetite
6. Chest pain
7. Loss of weight
8. General weakness
   - - 1. When do we say a person has active pulmonary TB? ( you can check more than one)
   1. When we have 2 or 3 positive smear tests
   2. one positive smear and positive X-ray
   3. Only chest positive x-ray
      - 1. When do we say a person has relapse TB? ( you can check more than one)
9. Completed treatment, cured and returned with positive smear
10. Under treatment, sputum remained positive after 5 months
11. Interrupted treatment for 3 months, returned with positive smear
12. Others Please specify:___________________________________
    - - 1. What is multi drug resistant TB or MDR-TB? ( you can check more than one)
13. it is when the bacillus is resistant to all currently available drugs to treat TB
14. it is when the bacillus is resistant to at least isoniazid and pyrazinamide
15. it is when the bacillus is very aggressive and you need at least 8 to 12 months of treatment
16. others please specify_____________________________

## Tuberculosis treatment

- 1. How long should be a new active pulmonary TB treated? (check only one)

1. 6 months
2. 9 months
3. 2-5 months
4. Don’t know
   1. How long (in months ) treatment of a person with PTB receiving intensive phase? (check only one)
5. 2 months
6. 6 months
7. 9 months
8. 12 months
9. Others please specify____________________

**Attitude towards TB control system** (check only one for each question)

- 1. New cases of TB are major challenge for TB control?

1. Strongly agree
2. Agree
3. Neutral
4. Disagree
5. Strongly disagree
   1. It is important to take more action to involve the community in the prevention and control of tuberculosis?
   2. Strongly agree
   3. Agree
   4. Neutral
   5. Disagree
   6. Strongly disagree
   7. TB patients often find it difficult to understand why they have to keep taking pills after starting to feel better
6. Strongly agree
7. Agree
8. Neutral
9. Disagree
10. Strongly disagree
    1. There is a difference in in treatment compliance by the patient if administered under DOTS?
11. Strongly agree
12. Agree
13. Neutral
14. Disagree
15. Strongly disagree
    1. Resistant tuberculosis is a major public health problem in this community?
       - 1. Strongly agree
         2. Agree
         3. Neutral
         4. Disagree
         5. Strongly disagree
    2. A person having TB in this community often faces a significant stigma and shame?
16. Strongly agree
17. Agree
18. Neutral
19. Disagree
20. Strongly disagree
    1. The way a person having TB receive their TB pills should take into account the individual circumstances of each person?
21. Strongly agree
22. Agree
23. Neutral
24. Disagree
25. Strongly disagree
    1. The lack adequate knowledge of the community about TB makes it difficult for a person to seek treatment for TB?
26. Strongly agree
27. Agree
28. Neutral
29. Disagree
30. Strongly disagree
    1. TB treatment we use is acceptable by our clients?
31. Strongly agree
32. Agree
33. Neutral
34. Disagree
35. Strongly disagree
    1. Most staff at your facility has adequate training for its activity?
36. Strongly agree
37. Agree
38. Neutral
39. Disagree
40. Strongly disagree

**Perceived Stigma towards TB (check only one for each question)**

- 1. Which statement is closest to feelings you have about a person having TB?

1. I feel compassion and desire to help
2. I feel compassion but I tend to stay away from this people
3. It is their problem and I cannot get TB
4. I feel fear because they may infect me
5. I have no particular feelings
6. Others please specify:______________________
   1. In your community, how is a person having TB usually regarded/treated?
7. Most people reject him or her
8. Most people are friendly, but they generally try to avoid him/her
9. The community mostly supports and help him/her
10. Others please specify: ___________________
    1. Do you think TB is a shameful disease?
11. Yes
12. No
    1. How do you feel when you are around a person having TB?
    2. I feel like I would get infected so I will make my conversation short
    3. I feel like I have to keep my distance
    4. I feel like I have to be supportive
    5. Others please specify:______________________

**Perception about Traditional healers (THs) and their practice**

1. Do you believe that traditional medicine exists?
2. Yes
3. No
4. Do you think people in this community go to traditional healers to get treated from TB?
5. Yes
6. No
7. If yes to Q2, which one do you think is more preferable? (check only one)
8. traditional medicine
9. Modern medicine
10. If traditional medicine is preferable, what do you think is the reason? ( you can check more than one)
11. easily accessible
12. traditionally acceptable
13. less time taking
14. others, please specify_________________________
15. Whom does the community prefer to consult for their health problems most of the time? (check only one)
16. Traditional healers
17. Trained health providers
18. If traditional healers are preferred, what do you think is the reason? ( you can check more than one)
19. Can be trusted more
20. easily accessible
21. no language barriers
22. have effective medication
23. religiously acceptable
24. others please specify__________
25. What do you think are the reasons that THs do not refer patients? ( you can check more than one)
26. THs can treat TB
27. THs fear losing patients’ trust
28. No referal mechanisms
29. THPs fear losing money
30. No referral system
31. No trust in modern medicine
32. THs fear of critics
33. Do not know
34. Others please specify: _______________
35. Do you accept traditional Health care practice?
    1. Yes
    2. No
36. Have you ever visited THs?
37. Yes
38. No
39. If yes to Q8, Which kind of traditional healers do they prefer to visit?
40. Healers who predominantly diagnose and treat by casting spells
41. Healers who treat with plants
42. Faith healers who use prayer and holy water
43. others please specify ___________________
44. Which health care service do you prefer?
45. Modern
46. Traditional
47. Both
48. What do you think are the advantage of traditional medicine? ( you can check more than one)
    1. Cheap and accessible
    2. Acceptable by community in popular
    3. Used for disease not managed by modern drugs
    4. Needs minimal training
    5. No advantage over modern medicine
49. In your opinion what are the disadvantage of THs? ( you can check more than one)
50. Lack of knowledge of determining drugs
51. No knowledge of scientific disease processing
52. Toxicity is common with their use
53. They are liable to be contaminated
54. No knowledge of contra indication
55. Unreliable diagnostic techniques
56. Have doubtful efficacy
57. Do you support collaboration of modern and traditional health practitioners, and integrating of the two systems on TB control?
58. Yes
59. No
60. What are the solutions you sought for the improvement of THPs? ( you can check more than one)
61. Scientific research
62. Training THs
63. Government assistance to TMPs
64. Provision of license to THs
65. Others please specify: ____________
66. Are you willing to work in collaboration with THs on TB control?
67. Yes
68. No
69. What are the possible way of collaboration with traditional Healers?( ( you can check more than one)
70. Cross visiting
71. Learning about traditional medicine
72. Working together
73. Traditional healers refer patients to health facility
74. Training THs
75. Joint research programs
76. Cross referral
77. Others please specify: _________
